# Offensive efficiency and traditional positional roles in Hungarian basketball: an empirical analysis

**DOI:** 10.3389/fspor.2025.1658662

**Published:** 2025-09-24

**Authors:** Botond Ágoston Nagy, Benedek Ágost Nagy, Ágoston Nagy, József Gáll, Tamás Sterbenz

**Affiliations:** ^1^ School of Doctoral Studies, Hungarian University of Sports Science, Budapest, Hungary; ^2^ Institute of Sport Sciences, University of Debrecen, Debrecen, Hungary; ^3^ Department of Applied Mathematics and Probability Theory, Faculty of Informatics, University of Debrecen, Debrecen, Hungary; ^4^ Sport Economics and Decision Making Research Centre, Hungarian University of Sports Science, Budapest, Hungary

**Keywords:** competition, efficiency, performance, talent, game analysis

## Abstract

**Introduction:**

This study explores the evolving offensive roles in professional basketball, focusing on the 2021/22 season of the Hungarian men's NB I/A championship. The primary aim is to analyze shifts in traditional positional responsibilities and compare offensive efficiency among Hungarian-educated players, import players, and young Hungarian (U23) players.

**Methods:**

A quantitative research design was applied to assess offensive performance across 239 player profiles, using official league statistics. Offensive efficiency was evaluated through multiple shooting efficiency metrics. Statistical analysis was conducted with IBM SPSS Statistics version 28.0, using ANOVA to detect significant differences among the groups.

**Results:**

Inside players showed higher shooting efficiency than positional averages. Import players consistently recorded the highest values across the analyzed indicators, followed by Hungarian-educated and U23 players. Notably, both domestic groups demonstrated the ability to take offensive responsibility, especially in decision-making situations such as passing to open teammates—often import players—who successfully completed possessions.

**Discussion/Conclusion:**

The findings suggest a shift in the function of traditional offensive positions, underscoring the superior efficiency of import players. Nonetheless, the active involvement of domestic players highlights developmental potential. These results support the need for increased investment in talent identification and development to strengthen domestic player performance and long-term competitiveness.

## 1 Introduction

Nowadays, basketball statistics, especially the analysis of advanced statistical indicators, play a decisive role in evaluating the performance of teams and players at different levels. Many questions can arise in the life of clubs, on which such analyzes can have a great impact. In most of the team sports, the final score is the main outcome of a match, but individual players are then put in the spotlight as their personal statistics are displayed. Nevertheless, everyone realizes that the “star players” would be ineffective without the support of their teammates (1). When building the team roster, we can get detailed feedback on the true role, value and performance of individuals. Preparing for the next match involves creating tactical plans, but the biggest challenge is selecting the right players. This process is complex, requiring careful decision-making by coaches to ensure victory. Success may primarily depend on the combination of different players and abilities, and to what extent they are able to fulfill the needs of the positions (2).

Previous research mostly focused on the technical, tactical and physical performance of players based on traditional positions (3–5), distinguishing between winners and losers (6–8), successful and unsuccessful teams (9, 10), starters and non-starters (11). The findings of Pojskić et al. (12) suggest that aerobic and anaerobic power and capacities can be good discriminative variables between players with different positional roles. The guards and forwards had a shorter recovery time and ability to efficiently repeat high intensity basketball-specific activities, while centers could play more powerfully. Nagy (13) also emphasized the importance of physical preparation who found that as the number of ball possessions and points shot from fast break increased on a team level, the number of high intensity sprints also increased per player. Sampaio et al. (3) found that forwards produced higher shooting efficiency from the paint area, which contributes more to the outcome of matches than the efficiency of guards and centers. Courel-Ibáñez & Suárez-Cadenas & Cárdenas-Vélez (14) investigated passes towards the rim in the NBA, and their research confirms that of Fewell et al. (15) as well, according to which the ball movement of NBA teams is mainly controlled by the point guard and secondarily by the shooting guard, while the power forward was primarily a finisher, and the centers usually have the highest success/failure ratio.

However, if we think of players such as LeBron James, Nikola Jokic or Luka Doncic, who in addition to their names, several positions appear during their characterization. Today's players can perform several positions and responsibilities thanks to their conditional or coordination skills. They have skills that make it impossible to classify them in a traditional position (16). Which means that the tasks and roles of the players have changed and are constantly changing. Various studies deal with this issue, and the five traditional posts have been replaced by the appearance of new positions. Kalman and Bosch (16) analyzed the NBA in this way and identified nine new positions. Wang et al. (17) created nine domestic and five import player positions by examining the Chinese Basketball Championship. Bianchi & Facchinetti & Zuccolotto (18), when studying the NBA, seven positions were created, while six were created in the Euroleague. Alagappan (19) mapped 13 positions when analyzing NBA players. Duman & Sennaroğlu & Tuzkaya (20) retained the five traditional positions in their study, but within these positions they established 4-4-4-5-6 different styles that suit players. Chen et al. (21) applied k-means clustering to play-type data from the Chinese Basketball Association (CBA) and identified fourteen offensive roles for domestic players and five for foreign players, demonstrating that specific clusters—such as 'Spot-up Wings who Attack' and “Bigs who Cut to the Rim”. These were significantly associated with team performance. Yamada and Fujii (22) introduced novel clustering methods for analyzing offensive lineups, combining shooting style distributions with offensive role classifications. Their results showed how lineup efficiency and player compatibility can be better understood through role-based and machine learning approaches, further supporting the shift away from the traditional positions.

Offensive effectiveness is at the center of our research, which is why we dealt with the relevant effectiveness variables from the data available to us. All of the mentioned researches used statistical and advanced statistical indicators proven in basketball in the formation of the new groups, positions, styles.

This study examines the 2021/22 season of the Hungarian men's NB I/A group championship. The uniqueness of the sample is defined by a rule (23) that is perhaps unique in the world in terms of professional championships. According to the rule, at least one Hungarian-educated player under the age of 23 must be on the court in the first half of each match. The introduction of the rule was a decision of the Hungarian Basketball Federation. As emphasized by the director responsible for youth development, clubs in the NB I/A league should adopt a long-term strategy for integrating youth players into senior teams, with the recently introduced regulation supporting this process by strengthening local identity and providing young players with a clear pathway to the first team (24). This rule is very interesting in itself, since Kalén et al. (25) observed that as players get older, their offensive activity changes, which is also related to positions. It has been shown that players in different positions have different periods of peak performance in their careers. For players in the inside position, this period is due later and lasts longer. That is one more reason why the talent identification and development have become extremely relevant in sports performance (26). Most of the scientific researches discuss the longitudinal and non-linear talent identification and development processes, the players’ qualities that determine their performances, and how the coaches could help the development of these promising athletes through the sports system (27). There are also a great amount of researches that try to determine who is considered as a talent, while relative age (28), growth, maturation, training age (29, 30) are taken into account. The scientific and non-scientific interest about talent identification and development does not look to stop in the near future, yet the applied and theoretical talent identification models have a low predictive value (31).

The 2021/22 season was selected for analysis due to the unique U23 player regulation and the completeness of statistical data available for that year. Additionally, league regulations allow a maximum of five foreign players per team, with no more than four permitted on the court at the same time, which also shapes team composition and playing opportunities. The aim of the research is to reveal the extent to which the traditional offensive role is changing. We would like to compare the offensive efficiency of Hungarian-educated, import, and young Hungarian (U23) players. Our goal is to get one step closer to being the first in Hungary to define the new roles and positions of the players.

In line with this, our primary intention was to conduct an exploratory analysis of offensive efficiency by examining all relevant indicators without imposing strong *a priori* assumptions. As described later in Section 2.3 Variable Selection, from 127 available variables we carefully selected 17 indicators based on literature review and their direct connection to offensive efficiency. By applying ANOVA, we tested the effects of positions, eligibility groups, and their interactions. Thus, rather than formulating narrowly defined hypotheses, our approach was to let the statistical outcomes determine whether differences exist, and if so, in which indicators.

Therefore, our general hypothesis was that (a) positions, (b) statuses (eligibility groups), or (c) their interaction could exert a significant influence on offensive efficiency. This means that in complex cases, the main and interaction effects must both be considered, since positions and statuses (eligibility groups) cannot always be meaningfully separated.

We assumed that the comparison of traditional positions and the three statuses (Hungarian-educated, Hungarian U23 and import) would not show large differences in efficiency indicators, but this assumption was intentionally kept broad to allow the data to reveal possible patterns.

## 2 Materials and methods

### 2.1 Data collection and pre-processing

The data was provided by the InStat (32) video analysis software and Fullcourt, the official basketball statistical program of the Hungarian championships. The data used in this study were provided by InStat and Fullcourt, where professional staff are responsible for coding and annotating all game events. In the case of Fullcourt, staff members are required to pass official exams and record live statistics on site during the matches, while InStat experts produce their statistics retrospectively through detailed video analysis. Thus, the statistical variables were not calculated by the authors themselves but obtained directly from these official sources. To ensure accuracy after data import, we conducted additional quality control by performing random video-based verification of approximately 5%–10% of the plays, as well as intermittent cross-checking of selected variables. This procedure confirmed the reliability of the dataset and minimized the potential risk of import errors. In our research, we examined the 2021/2022 season of the Hungarian NB I/A group championship. The sample included 239 player profiles. The profiles were classified into three statuses (also known as eligibility groups). Players who appear in the league as foreign nationals were placed in the import status. The second is the status of Hungarian-educated players. Players older than the age of 23 competing as Hungarian citizens belong to this category and the import players who were competing in the Hungarian leagues and spent 30 months before the age of 21. The third is the Hungarian U23 status, who play as Hungarian citizens and were born in 1999 or later in the given championship year. Based on these, there were 93 import, 60 Hungarian-educated and 86 Hungarian U23 players in the database. It is important to draw attention to the fact that these classifications are provided by the Hungarian Basketball Federation and is established in the competition system (23).

Each player had one full league season in the sample. In total, there was a transfer during the season in one case. In order to obtain more accurate results and avoid bias, we narrowed down the sample. The narrowing criteria are a minimum of 5 matches played, and an average of at least 5 min spent on the court (17, 20), those who didn't meet these conditions were excluded, thus we got 194 of the 239 players. All three statuses are represented by a sufficient number of elements.

### 2.2 Traditional positions

The general characterization of traditional positions was provided by Bianchi & Facchinetti & Zuccolotto (18), who attempted to formulate it in their research. The official website of the NB I/A championship can be found on the website of the Hungarian Basketball Federation. In addition to the statistics of all players, their playing positions are also displayed. It is important to emphasize that these positions are designated by the teams, specifically by the coaching staff. In the Federation's database, instead of the well-known 5 traditional positions the teams provided 4 more positions in addition, representing the combinations of the original five positions (33). These nine positions were collected and summarized in Table 1.

**Table 1:** Traditional positions and their definition (18) complemented by the four additional positions provided on the official website of the Hungarian basketball federation (33).

Traditional positions	
1—Point Guard or Playmaker	He is intended to be the “brain” of the team, the player who “creates the game”; the Point Guard is often the shortest player in the team.
2—Shooting Guard	Usually, the main duty of a Shooting Guard is to score points far from the basket; there are exceptions represented by defensive minded players, whose role in the team is to stop the best offensive player of the opposite team.
3—Small Forward	Small Forwards are usually very athletic players who can score from different areas of the ﬁeld; they also help in defending and rebounding.
4—Power Forward	Power Forwards are expected to play closer to the basket, mainly helping the team by scoring points and taking rebounds.
5—Center	The Center is supposed to be the biggest player in the team, the one who grabs a lot of rebounds, protects the rim on defense, blocking shots and taking advantage of his size on offense.
1–2	This position category was defined by the coaching staff and likely encompasses, or even combines, the roles of the point guard (1) and shooting guard (2). It can also be assumed that, depending on lineups and playing time in a given match, players may appear across multiple roles.
2–3	This position category was defined by the coaching staff and likely encompasses, or even combines, the roles of the shooting guard (2) and small forward (3). It can also be assumed that, depending on lineups and playing time in a given match, players may appear across multiple roles.
3–4	This position category was defined by the coaching staff and likely encompasses, or even combines, the roles of the small forward (3) and power forward (4). It can also be assumed that, depending on lineups and playing time in a given match, players may appear across multiple roles.
4–5	This position category was defined by the coaching staff and likely encompasses, or even combines, the roles of the power forward (4) and center (5). It can also be assumed that, depending on lineups and playing time in a given match, players may appear across multiple roles.

Recent research has highlighted that traditional five-position classifications may no longer adequately reflect modern basketball. For example, Péndola-Reinecke et al. (34) applied a data-driven clustering approach (PCA and k-means) in women's basketball, identifying three new role-based categories—perimeter specialists, defensive specialists, and primary scorers/rebounders. These results underline the importance of role-based, dynamic classifications and support the need for empirical examinations such as the present study.

### 2.3 Variable selection

Considering the specialty of the Hungarian championship, we started the examination of the sample with the statistical analysis of the performance indicators deemed significant based on the preliminary statistical results. In line with this, the process involved three steps: (i) establishing a general rationale, (ii) filtering variables unrelated to offensive efficiency, and (iii) selecting the final set of 17 indicators.

From the available 127 variables, after processing the literature, we examined a total of 17 variables, which are provided by basketball statistical indicators and advanced statistical indicators (Table 2). Each metric is directly or indirectly related to the efficiency and performance of the players. The indicators that are not included in the present study were those that did not directly connect to the offensive efficiency. Among the traditional statistics, this applies for example to steals, blocked shots, and defensive rebounds. In addition, several play-type indicators were excluded as they primarily describe offensive and defensive actions (e.g., catch and shoot, cuts, drives, hand-off, isolation). Furthermore, individual offensive measures such as successful two-point or three-point shots were not analyzed separately, since efficiency-based metrics like EFG% and TS% already encompass these outcomes.

**Table 2:** Basketball statistical indicators and corresponding notations.

Basketball statistical indicators	
Games	Games played
PtsTot	Total points scored by a player during the season
PPPP	The player's points scored from ball possessions on average
FGATot	A player's total field goal attempts during the season
FTMTot	All made free throws by a player during the season
OffRtg	Offensive Rating (by Dean Oliver) — The team's points scored per 100 possessions when the player was on the court
DefRtg	Defensive Rating (by Dean Oliver) — The team's allowed points per 100 possessions when the player was on the court
PM	The team's point difference in total during the player's time on the court
NetRtg	Net Rating (by Dean Oliver) — The difference between the team's points scored and allowed per 100 possessions when the player was on the court
AsTo	Ratio number, assists divided by the turnovers
StTo	Ratio number, steals divided by turnovers
TS%	True Shooting Percentage — A measure of shooting efficiency that takes into account 2-point field goals, 3-point field goals and free throws
EFG%	Effective Field Goal Percentage (by Dean Oliver) — This statistical metric adjusts for the fact that a 3-point field goal is worth one more point than a 2-point field goal
USG%	Usage Percentage — Usage percentage is an estimate of what percentage of the team's possessions a player used while on the field
FTF	Free throw factor (by Dean Oliver), made free throw attempts divided by all field goal attempts
PtsAv	A player's average points scored during the season
PMAv	During the player's time on the court, the team's point difference is average

### 2.4 Statistical analysis

First, we analyzed the specific “performance” variables by simple descriptive statistics (in particular sample mean and median, standard deviation, maximum and minimum to show their range). Then the main goal of our statistical analysis was to compare the different groups of players based on two grouping variables, namely the “position”—which gives a classification in 9 groups— and the “status” of the players—which classifies players into 3 groups. For the comparison of means of several performance variables we applied two-way analysis of variance (ANOVA), such that interactions of the two grouping variables were also included in the model. Afterwards, we have also run *post-hoc* tests, namely Tukey tests for a more detailed pairwise comparison of positions and statuses.

The ANOVA tests are known to be fairly robust regarding the failure of some assumptions (normality, equal variances), though we are aware of the fact that one needs to be more careful to read the results in certain cases of ours, in particular, where in a few categories the (sub)sample sizes are low (hence asymptotic statistical properties may not hold yet). Thus, on the one hand we focus only to the fairly significant differences, on the other hand –as a pragmatic threshold– we flagged any subgroup representing less than 2,5% of the total sample.

For all analysis we used IBM SPSS Statistics version 28.0.

## 3 Results

### 3.1 Distribution of players with respect to position and status

During the preliminary processing, the players were classified into nine positions and three statuses.

The next table shows the joint distribution of players (in % of total after the exclusion of the sample) with respect to the two grouping variables. Furthermore, the last column and row gives this way the percentage frequency distribution with respect to each grouping variable separately. The players who didn't play in at least 5 games and didn't average at least 5 min were excluded from the sample (Table 3). Table 3 shows the crosstabulation with respect to position and status after exclusion of the sample.

**Table 3:** Crosstabulation with respect to position and status after filtering in the sample (% of total).

The positions and statuses in the Hungarian League				
Positions	Status			
	Hungarian U23	Hungarian educated	Import	Total
1	4,1%	3,1%	6,7%	13,9%
1–2	5,2%	3,6%	5,2%	13,9%
2	3,1%	1,5%	5,2%	9,8%
2–3	2,6%	2,6%	6,2%	11,3%
3	5,2%	2,6%	3,6%	11,3%
3–4	1,0%	3,1%	4,6%	8,8%
4	3,1%	2,1%	3,6%	8,8%
4–5	1,0%	2,6%	5,7%	9,3%
5	3,6%	4,1%	5,2%	12,9%
Total	28,9%	25,3%	45,9%	100,0%

The highest number of positions 1 (13,9%—27 players), 1–2 (13,9%—27) and 5 (12,9%—25) occurred in the championship, while the lowest number is for positions 3–4 (8,8%—17), 4 (8,8%—17) and 4–5 (9,3%—18).

The highest proportion was imported players (45,9%—89), followed by Hungarian U23 players (28,9%—56), then Hungarian-educated players (25,3%—49).

To preliminarily characterize the sample, several indicators widely applied by coaches and in international practice are presented in Table 4. After applying the filtering (exclusion) criteria, the descriptive statistics reveal clear differences between the three groups. Hungarian U23 players show the lowest averages across all indicators, in points scored (M = 62,93) and shooting efficiency (TS% = 48,32; EFG% = 49,62), with very low usage (USG% = 0,12). Hungarian-educated players present intermediate values in most categories (PtsTot M = 204,40; TS% = 49,58; EFG% = 52,11; USG% = 0,16), suggesting a more balanced role within teams. Import players stand out with the highest point production (M = 324,13) and shooting efficiency (TS% = 54,31; EFG% = 57,72), combined with the highest usage (USG% = 0,22). Offensive Ratings are relatively similar across the three groups (U23: 92,98; Hungarian-educated: 95,12; Import: 96,28), while Net Ratings are slightly negative in all cases (U23: −4,81; Hungarian-educated: −2,18; Import: −0,53). Age differences follow expectations: U23 players are the youngest (M = 21,23), while Hungarian-educated (M = 28,88) and Import players (M = 28,68) are significantly older.

**Table 4:** Descriptive statistics of the players, including the total points scored (ptsTot), offensive rating (offRtg), Net rating (netRtg), true shooting percentage (TS%), effective field goal percentage (EFG%), usage percentage (USG%), Age (SD: standard deviation).

Descriptive statistics of the sample								
Status	Descriptives	PtsTot	OffRtg	NetRtg	TS%	EFG%	USG%	Age
Hungarian U23	Mean	62,93	92,98	−4,81	48,32	49,62	0,12	21,23
	Max	470,00	107,00	17,00	83,33	83,33	0,23	23,00
	Min	0,00	73,00	−36,00	0,00	0,00	0,04	17,00
	Median	37,00	94,00	−4,00	50,00	50,87	0,12	22,00
	SD	77,62	7,03	11,04	14,25	13,95	0,04	1,40
Hungarian-educated	Mean	204,40	95,12	−2,18	49,58	52,11	0,16	28,88
	Max	801,00	108,00	22,00	67,66	67,97	0,29	37,00
	Min	8,00	76,00	−44,00	12,50	20,19	0,09	24,00
	Median	148,00	95,00	−1,50	52,44	53,71	0,16	28,00
	SD	185,36	6,64	11,22	10,75	9,84	0,05	4,07
Import	Mean	324,13	96,28	−0,53	54,31	57,72	0,22	28,68
	Max	756,00	110,00	15,00	76,19	69,73	0,31	37,00
	Min	36,00	87,00	−13,00	39,47	43,35	0,11	22,00
	Median	318,00	96,00	−2,00	53,95	57,94	0,22	29,00
	SD	177,40	3,82	5,74	6,04	5,28	0,04	3,03

### 3.2 Differences and impacts of positions and statuses

#### 3.2.1 The role of positions

Concerning the nine (1, 2, 3, 4, 5, 1–2, 2–3, 3–4, 4–5) positions, a significant difference was found by ANOVA for six indicators (Table 5). These metrics include points/player's possessions; ratio of assists to turnovers; ratio of steals to turnovers; the true shooting percentage; the effective field goal percentage and usage percentage. Furthermore, based on the pairwise comparison of positions by the Tukey tests, Table 6, Part A presents the homogeneous subsets identified by the test for all indicators where statistically significant differences occurred (at significance level of 5%).

– **Point/Player Possession (PPPP)**
  – We found differences at positions 4, 5, 3–4, 4–5.
  – All of the mentioned positions produced an average of at least 0,95 points. Position 4: 0,9959; 5: 0,9980; 3–4: 0,9535; 4–5: 0,9867. In contrast, the other positions performed below the post average (0,9192). Position 1 produced the fewest points with 0,8530.
  – **Ratio of assists to turnovers (AsTo)**
  – There were two positions that didn't reach the 1,0 indicator. Players in positions 5 and 4–5 had, on average, more turnovers than assists. The value of position 5: 0,767, while position 4–5: 0,901. Two more positions (2: 1,427; 3: 1,233) didn't reach the position average (1,448).
  – The highest value was shown by those in position 1 with 1,913. Position 1–2 also had an outstanding value: 1,774, and position 4: 1,742.
  – Although several significant differences were identified, the most pronounced one –showing the largest mean difference– is that position 5 significantly differs from positions 1, 4, and 1–2 by the Tukey test.
– **Ratio of steals to turnovers (StTo)**
  – The players in position 3–4 showed the most significant difference in the ratio of steals to turnovers. Only this position achieved an average above 1,0, exactly 1,064.
  – Position 2 (0,756) and 4 (0,788) show noteworthy values, but position 5 achieved the lowest value: 0,437.
  – Among the significant different pairs the most pronounced one (with the largest mean difference) is that position 3–4 significantly differs from positions 5 by the Tukey test.
– **True shooting percentage (TS%)**
  – The players in the traditional sense of the inside positions achieved a higher percentage. The players with positions 4 (56,15%), 5 (58,59%), 3–4 (54,94%), and 4–5 (57,44%) completed above the average of the positions (53,79%).
  – The lowest value was achieved by position 2, 50,04%.
  – A clear significant difference was identified between position 5 and position 2 by the Tukey test.
– **Effective filed goal percentage (EFG%)**
  – The average of the positions was 51,12%. Four positions performed above that. Position 5 has the highest value, 57,01%. This is followed by position 4–5, 54,98%, then position 4 (53,89%) and 3–4 (53,25%).
  – The perimeter positions in the traditional sense performed below average, and the lowest indicator was achieved by position 2 (47,03%).
  – Significant differences were identified between position 5 and either position 2 or 2–3 by the Tukey test.
– **Usage percentage (USG%)**
  – Position 1 (19,73%) and 4–5 (19,59%) reached the highest values. While the lowest was held by position 3 (15,74%) and 4 (15,76%). The average usage percentage of the positions was 17,88%.
  – The largest mean differences are given between positions 4–5 or 1 and positions 3 or 4, which pairs are all significantly different according to the Tukey test.
– **Summary of position results**
  – For those metrics that focused on shooting efficiency (PPPP, TS% EFG%), interior position players (4, 5, 3–4, 4–5) performed above the position average. The lowest value was achieved by the players in position 2 in the case of TS% and EFG%, while they showed the second lowest value in the PPPP index.
  – Position 1 performed the best in the AsTo (1,913), and this may also be related to the USG%, where position 1 also achieved the highest value (19,73%).
  – It's an interesting result that when looking at the StTo, only position 3–4 reached a value above 1,0, exactly 1,064. Pairing this result with USG% speaks volumes, since position 3–4 has the third lowest indicator (16,7%). Only position 3 (15,7%) and 4 (15,8%) showed a lower percentage.
  – For the indicators that focus on turnovers (AsTo, StTo), position 5 performed the weakest with 0,77 and 0,44.

**Table 5:** Simplified table of *P*-values of the two-way ANOVA with respect to positions and statuses; *, ** denotes significant cases at level 0,01 and 0,001, respectively, gray background refers to * and ** cases.

Differences within the statuses									
Player classification	Games	PtsTot	PPPP	FGATot	FTMTot	OffRtg	DefRtg	PM	NetRtg
Pos	0,632	0,662	<0,001**	0,435	0,663	0,813	0,659	0,981	0,493
Status	0,005*	<0,001**	<0,001**	<0,001**	<0,001**	<0,001**	0,922	0,209	0,007*
Pos & Status	0,136	0,404	0,062	0,228	0,519	0,188	0,777	0,990	0,421
Player classification	AsTo	StTo	TS%	EFG%	USG%	FTF	PtsAv	PMAv	
Pos	<0,001**	<0,001**	0,004*	<0,001**	0,003*	0,079	0,176	0,937	
Status	0,446	0,358	<0,001**	0,006*	<0,001**	<0,001**	<0,001**	0,384	
Pos & Status	0,005*	0,048*	0,037*	0,071	0,140	0,370	0,107	0,994	

**Table 6:** Results of *post hoc* Tukey tests (with sig. level 5%) showing the homogeneous subsets of positions **(A)** and statuses **(B)**. U23, Hungarian U23; HE, Hungarian-educated; IP, =import players. Status and position classes in the subsets are listed in increasing order w.r.t. sample mean.

Results of *post hoc* Tukey Tests: Positions and Statuses					
A: Homogeneous Subsets for the Positions					
AsTo	StTo	TS%	EFG%	USG%	
{5,45,3,2}, {45,3,2,23,34}, {3,2,23,34,4,12,1}	{5,45,1,12,3,23,2}, {45,1,12,3,23,2,4}, {2,4,34}	{2,23,1,12,3,34,4,45}, {23,1,12,3,34,4,45,5}	{2,23,1,12,3,34,4,45}, {1,12,3,34,4,45,5}	{3,4,34,23,2,5,12}, {34,23,2,5,12,45,1}	
B: Homogeneous Subsets for the Statuses					
Games	PtsTot	PPPP	FGAtot	FTMTot	OffRtg
{U23, PI}, {HE}	{U23}, {HE}, {IP}	{U23, HE}, {IP}	{U23}, {HE}, {IP}	{U23}, {HE}, IP}	{U23, HE}, {HE, IP}
NetRtg	TS%	EFG%	USG%	FTF	PtsAv
{U23, HE}, {HE, IP}	{U23, HE}, {IP}	{U23, HE}, {IP}	{U23}, {HE}, {IP}	{U23}, {HE}, {IP}	{U23}, {HE}, {IP}

#### 3.2.2 The role of statuses

Regarding the statuses (Hungarian U23, Hungarian-educated, import), we found significant differences by ANOVA in 12 out of 17 indicators (Table 5). These indicators are: number of games, total points scored, points/player's possessions, total field goal attempts, total made free throws, offensive rating, net rating, true shooting percentage, effective field goal percentage, usage percentage, free throw factor, average points scored. Table 6, Part B presents the homogeneous subsets identified by the *post hoc* Tukey tests with respect to statuses, highlighting all cases of indicators where statistically significant differences occurred by the test.

– **Number of games (Games)**
  – The Hungarian-educated players took to the field the highest average, followed by the group of imported players, and then the Hungarian U23 players. In the league, the players played in an average of 26,32 matches. On average, Hungarian youths got on the court in 24,32 matches, domestically educated ones in 30,61 matches (significantly different by the Tukey test from the other two statuses), and imported players in 25,21 matches.
– **Total points scored (PtsTot)**
  – There was a significant difference between the three statuses. The imports scored the most points, averaging 328,65 points. In contrast, the other two statuses remained below the league average (222,44 points). The Hungarian-educated players produced 204,86 points, while the U23 players scored only 69,04 points. Note that all the three statuses are significantly different by the Tukey test.
– **Points/player's possessions**
  – The foreigners performed significantly better in this indicator, scoring 0,9888 points per possession (significantly different by the Tukey test from the other two statuses), which exceeds the league average (0,9192). The Hungarians produced 0,8812 points, while the U23-s produced 0,8418 points per possession.
– **Total field goal attempts (FGATot)**
  – The championship average in the number of field goal attempts was 174,90. Only import players had more attempts on average, 252,00. The group of young players falls far short of this with an average of 60,39 attempts, while the Hungarians came close (165,71), but do not reach the average. Means of all the three statuses are significantly different by the Tukey test.
– **Total made free throws (FTMTot)**
  – The foreign group had almost twice as many successful free throws (58,84) as the Hungarian-educated status (30,04). The U23 players (8,29) were far below the league average (36,97). Here again, means of all the three statuses are significantly different by the Tukey test.
– **Offensive Rating (OffRtg)**
  – The examination of the index of points generated from 100 ball possessions brought a significant difference between the statuses. The Hungarian and foreign players performed above the average (U23: 92,94; Hungarian: 95,22; Import: 96,39; Average: 95,10). The import and young statuses gave significantly different means by the Tukey test.
– **Net rating (NetRtg)**
  – All three statuses achieved results with a negative sign. The league average was −1,98 points. Two statuses performed better. Foreigners had −0,60 points, Hungarian-educated players had −1,64 points, while U23 players averaged −4,44 points. The import and young statuses were found to have significantly different means by the Tukey test.
– **True shooting percentage (TS%)**
  – The import player status performed best, with 57,22% (significantly different by the Tukey test from the other two statuses), while the other two statuses were lower than that (U23: 50,12% domestic: 51,75%). The average value of the championship was 53,79%.
– **Effective field goal percentage (EFG%)**
  – The average of the championship players was 51,12%. Only foreigners achieved a higher value, 53,76% (significantly different from the other two statuses by the Tukey test). The other two statuses were 48,67% (U23) and 49,13% (Hungarian-educated).
– **Usage percentage (USG%)**
  – The import player status was the most active on the court with a 22,25% indicator. This was followed by the Hungarian status with 16,09% and the U23-s with 12,52%. The average result of the league was 17,88%. All the three statuses found to have significantly different means by the Tukey test.
– **Free throw factor (FTF)**
  – The average FTF was 0,18. The lowest indicator was shown by the young status (0,12), with a higher result for the domestic-educated status (0,17) and then to foreigners (0,23). Note furthermore, that all the three statuses are significantly different in means by the Tukey test.
– **Average points scored (PtsAv)**
  – The average of the league was 8,08 scored points. Only a status of foreigners achieved it (12,85 points). Hungarian-educated people scored 5,84 points, while U23-s scored 2,46 points. All the three statuses are significantly different in means by the Tukey test.
– **Status Summary**
  – The foreigners achieved higher performance in all indicators except for game number. In game number index, Hungarian-educated players showed a significant difference compared to the other two statuses (Hungarian: 30,61; import: 25,21; U23: 24,32; average: 26,32).
  – The other 11 indicators showed a significant difference between the statuses. The highest value was given by import players, followed by the Hungarian-educated, followed by the U23 players.
  – Analysis of the Total Scored Points, PPPP, Total Field Goal Attempts, All Made Free Throws, USG%, Free Throw Factor, Average Scored Points detected that there was a significant difference between import players and Hungarian-educated players. In addition, there was another significant difference between the results of Hungarian-educated and young Hungarian U23 players.
  – In the case of OffRtg and NetRtg, the difference between imports and Hungarian players was not as significant as the results of the import players compared to the U23 young players.
  – The investigation by the TS% and the EFG% showed that the imports’ values were significantly overwhelmed by Hungarian-educated and U23 players. While the difference between the last two statuses was not that big.

#### 3.2.3 Combination of positions and statuses

Which concerns the joint impact of positions and statuses (i.e., the interactions in the ANOVA, see last line of Table 5), interactions were significant in case of 3 indicators, namely: the rate of assists to turnovers, the steals to turnovers ratio and the true shooting percentage.

It should be noted that due to the low (sub)sample sizes in certain subgroups one should omit to derive strong conclusions, since the results in these cases may not be statistically relevant. For this, we suggest a threshold of 2,5% of the total sample, according to what combinations U23: 3–4 and 4–5 and Hungarian educated 2 and 4 show lower sample size. Though, in what follows, for the sake of completeness we also mention these categories in the general discussion.

– **Ratio of assists to turnovers (AsTo)**
  – The AsTo ratio average for positions was 1,448. The average result of the statuses: for Hungarian U23 players 1,366; result of Hungarian-educated 1,583; while the import status belongs to 1,426.
  – The highest value was achieved by young players in the position 4, with a 2,652 value. Furthermore, in position 3–4 the young Hungarians (2,585) and Hungarian-educated players (2,217) show an outstanding result. Hungarian-educated 1-s (2,377) and import player 1-s (2,222) also have a significant high value.
  – In position 5, all three statuses performed below average. U23-s are 0,497; Hungarian-educated 0,829; Foreigners produced a ratio of 0,907. The 3–4 import status (0,981) also has a low value. In addition, the 4–5 young players were featured with the lowest (0,320) index and the 4–5 import status (0,953) performed poorly too.
  – Each group of position 1–2 achieved an average indicator (U23: 1,627 Hungarian: 1,883 imports: 1,846). Position 3 statuses performed slightly below average. The 2–3 Hungarian players caught up to the leaderboard with a 1,978 indicator.
– **Ratio of steals to turnovers (StTo)**
  – The average value of the positions of StTo was 0,661. The average of the statuses was: 0,658 for Hungarian U23 players; the result of Hungarian-educated is 0,718; while the import group was 0,631.
  – The highest value was reached by position 3–4, and each statuses exceeded the status average (U23: 1,335 Hungarian: 1,410 imports: 0,772). Young players in position 2 (1,088) also achieved a very high rate.
  – The top performers also include each status of the position 4 (0,747; 0,838; 0,794) and the position 2–3 from the young players (0,710) and the Hungarians (0,88). The foreign 3-s (0,786) and Hungarian 2-s (0,743) belong here as well. The 1–2 foreign status (0,697) is also noteworthy.
  – The lowest value was shown by the position 5-s. Each was below the average (U23: 0,387 Hungarian: 0,469 imports: 0,446). The lowest value was shown at status level by position 4–5 U23 players with a value of 0,345. The other two statuses also remained below average (Hungarian: 0,626 imports: 0,547). In addition, Hungarian-educated 3-s (0,468) and 1–2-s (0,539), U23 1-s (0,510) and import 2-s (0,56) and 2–3-s (0,589) also had poor results.
– **True shooting percentage (TS%)**
  – The average value of positions of TS% was 53,79%. The Hungarian U23 status was 50,12%, the Hungarian-educated players were 51,75% and the import players were 57,22%.
  – Each group of position 5 reached an outstanding percentage. Young players are 56,87%, Hungarians 54,79% and imports 62,83%. Young players in position 4 (58,30%) and Hungarians (56,95%) also produced high values compared to the statuses and positions. In addition, the 3–4 U23 players (61,35%), 4–5 Hungarians (58,22%) also reached a high number.
  – The weakest position was 2–3, where each statuses performed below the status average (U23: 45,70% Hungarian: 47,62% import: 54,68%). The lowest TS% was reached by the young status 2-s, with 36,57%. In addition, the Hungarian 1-s (45,48%), the 1–2 Hungarian position (47,76%) and the 4–5 young position (47,75%) were significantly below the average value. Import 4-s (53,86%), 2–3-s (54,68%), 3–4-s (55,68%) were also below the average of import status but performed above the average of the positions.
– **Positions and Statuses Summary**
– **Ratio of assists to turnovers (AsTo)**
  – Analyzing the statuses within the positions, the Hungarian U23 players always performed the weakest, except for position 2, 4 and 3–4. For the latter two, they performed best within the positions. In 5 of the 9 positions, the Hungarian-educated group reached the highest value (1, 2, 1–2, 2–3, 4–5).
– **Ratio of steals to turnovers (StTo)**
  – In 6 of the 9 positions, the Hungarian-educated players had the highest value (1, 4, 5, 2–3, 3–4, 4–5.
– **True shooting percentage (TS%)**
  – Comparing the statuses of the positions, except for position 4 and 3–4, the import status always reached the highest percentage (1, 2, 3, 5, 1–2, 2–3, 4–5).

Let us note finally that we are aware of the fact that certain measures (variables) of the players may not necessarily be (totally) independent, some weak dependence may occur whitin some sample elements. However, the ANOVA tests are known to be fairly robust, hence we believe that this limitation is not crucial regarding the main findings.

#### 3.2.4 Short explanation of non-significant indicators

In addition to the significant results, it is important to highlight those indicators for which no statistical differences were observed. For example, we did not find any significant differences in defensive rating (DefRtg), plus/minus (PM), or average plus/minus (PMAv) between positions, statuses, or their interaction. This lack of difference may highlight the fact that these indicators may happen on a collective, team-level dynamics than by individual positions or player statuses. It should also be emphasized that the aim of the present study was not to evaluate the technical or tactical decisions made by coaches and players, nor to examine the qualitative aspects of team strategies. Instead, our primary goal was to explore how offensive efficiency and position-status structures interact statistically, providing a framework for further research that could examine the practical and tactical dimensions in more detail.

## 4 Discussion

The aim of the present study was to reveal how much the traditional offensive role is changing. We wanted to compare the three different statuses that we created within the players in terms of offensive efficiency. We expected no big difference between the traditional positions and the three different statuses in terms of offensive efficiency indicators.

Our analysis is based on the nine-position classification created by the coaches working in the Hungarian League, instead of the traditional five-position classification. By introducing four additional categories to the Federation, the coaches expanded the classification system to nine positions. The main reason behind this classification could be that today's players are capable of performing and executing different tasks in different positions during the games. Moreover, these players can change their playing styles game by game depending on the upcoming match ups. Because of these adjustments new positions were born, so the players could be categorized more precisely (18, 19). These new positions could produce a better picture of the players and an understanding about the game and about the needs of a team (16, 17, 20–22, 34).

During the ANOVA we found six indicators that were significantly different with respect to positions. The metrics that focused on the shooting efficiency (PPPP, TS%, EFG%), the inside players (4, 5, 3–4, 4–5) reached higher values than the position average. While the lowest values were produced by position 2. This finding meets Sampaio et al. (3), who found that the paint area was a key factor in effectiveness, and the forwards reached higher values than the guards and centers. Position 1 reached the highest value in AsTo (1,913) and USG% (19,73%). This reflects very well on Courel-Ibáñez & Suárez-Cadenas & Cárdenas-Vélez (14) and Fewell et al. (15) findings, where the point guards and shooting guards are the main controllers of the ball movement and in the other hand the power forwards and centers are the primary finishers of the offense. Furthermore, the metrics that involve the turnovers position 5 performed the worst (0,77 and 0,44). The StTo paired up with the USG% gives us an interesting result. Only position 3–4 reached a value above one (1,064), while they had the third lowest value (16,7%) in USG%. This could mean that these players are very good in stealing the ball from the opponent, while they also take care of it in the offense. In addition, as we take a look at the scoring efficiency values, position 3–4 also reached higher values then the other positions. It means these types of players could be very good defenders and also very effective finishers, though they are not the main scorers. One possible explanation is their flexible tactical role, combining perimeter and inside responsibilities. However, since tactical and physical parameters were not part of the dataset, these interpretations remain speculative. It should be emphasized that these differences reflect associations rather than causal mechanisms. Our study was not designed to establish cause-and-effect relationships but to reveal statistical patterns in offensive efficiency.

Based on the ANOVA results with respect to the statuses we have found a simple pattern: in every index where the variable statuses were found significant (i.e., some statuses show significant differences), the import players had the best values, that was followed by the Hungarian-educated players and lastly the Hungarian young players. The only exception was the number of games played. In seven indices (PtsTot, PPPP, FGATot, FTMTot, USG%, FTF, PtsAv) there was a significant difference between import players and Hungarian-educated players and another significant difference between the results of Hungarian-educated and young Hungarian U23 players. In the case of OffRtg and NetRtg, the import players and Hungarians didn't show a big difference but their numbers compared to the U23 players showed a quite high difference. The TS% and the EFG% indices showed significant differences between the import players compared to the Hungarian-educated and U23 players. Kalén et al. (25) examined the offensive activity and the periods of peak performance. In reflecting to the results of Kalén, here in that study we also got a clear picture of the correlation between the offensive efficiency and the age.

Finally, in some cases — i.e., three indices — we obtained significant interactions between positions and statuses by the ANOVA. (Recall again that subgroups U23: 3–4 and 4–5, plus Hungarian educated 2 and 4 have sample sizes under 2,5% of total, hence we suggested to omit strong conclusions for these cases.) The AsTo showed that the Hungarian U23 players had the lowest values except for position 2, 4, 3–4. For the latter two, they performed best within the positions. The Hungarian-educated players in position 1, 2, 1–2, 2–3, 4–5 reached the highest values within the positions. That means the U23 Hungarian and Hungarian-educated players took responsibility in offense and could make good decisions when to pass the ball to an open teammate who could finish the possession with a basket. In 6 of the 9 positions, the Hungarian-educated players had the highest value (1, 4, 5, 2–3, 3–4, 4–5) in the StTo. That can strengthen the previous result, which means that the Hungarian-educated players can steal the ball and create a good look in the other side of the court. The third index is the TS%, where except for position 4 and 3–4, the import status always reached the highest percentage. When examining the assist-to-turnover ratio (AsTo), steal-to-turnover ratio (StTo), and true shooting percentage (TS%), significant differences emerged across positions, while the interaction of positions and statuses also proved to be meaningful. The status itself showed a significant effect only in the case of TS%, where import players showed higher shooting efficiency compared to Hungarian-educated and U23 players. These results suggest that the informative value of the indicators lies primarily considering position and status together, rather than comparing the players' statuses in isolation. Therefore, while differences in shooting efficiency between Hungarian and import players can indeed be observed, broader interpretations regarding offensive roles should be applied with caution. However, these results may provide a valuable basis for future research to explore how positional and status-related factors jointly create and shape the offensive responsibilities and player development pathways. Future research could extend these findings by including explanatory covariates such as tactical role, physical attributes, or career history. These could help identify the underlying mechanisms behind the observed statistical differences.

These findings underline that it is worth putting a lot more resources in the talent identification and development so the next generation players could grow up to their assigned tasks and perform on a level that the leaders, coaches and fans are expecting from them. On a high level where performance matters and where there are consequences, the process of predicting which athletes are most likely to succeed (i.e., international, national or regional competition) will always encompass risks and mistakes (35). With a more accurate talent identification and development system, we can prepare the players for success, which helps them achieve their goals, that is, win. The more information we have about the physical and mental resources, the more effective practice plan can be designed (36). Cabarkapa et al. (37) emphasized adjusting training to players' maturation status. Changing technologies, genes, mindset may help explaining why the athletes are getting stronger, faster, bolder, and better than ever (38). Ultimately, this is the common interest of all professionals interested in sports. While the dataset refers to the 2021/22 season, the observed patterns remain relevant due to the structural consistency of the league and the continued enforcement of key rules such as the U23 regulation.

## 5 Possible practical implications

• Traditional basketball positions are becoming more flexible, so teams need to develop players who are capable of fulfilling multiple roles on the court. For example, players may need to play as a 3-point specialist, dominant scorer, facilitator who focuses on creating high quality open shots for the teammates, versatile rebounder, stretch big who can score from close and long distances.
• More precise player categorization can help create personalized training programs that better reflect individual strengths and team needs. For instance, a tall and physically dominant player might need a different conditioning, shooting and technical program than a smaller, 3-point specialist, who most of the time operates on the perimeter and is exposed to less physical contact than inside players.
• In order to enhance talent identification and development, a promising way could be to pay more attention to accurate assessment of players’ physical and mental resources. For example, it could be beneficial if the Federation organized open development training camps in the off-season, providing a better picture on the necessary modifications or updates to training and education programs. Furthermore, nowadays it's increasingly recommended for the clubs to employ sports psychologists not only for professional senior teams but youth teams as well, to support their mental development from an early age through to the professional level. Although the availability of more resources may provide greater opportunities, it does not necessarily guarantee better training outcomes. The decisive factor remains the consistent implementation of high-quality professional work, which is essential for the effective development of players.
• Training sessions and tactical plans must adapt to new roles, such as the stronger defensive and offensive capabilities of Hungarian-educated players. For instance, targeted drills could emphasize the proper arm- and footwork for stealing the ball successfully from a ballhandler, or to take a charge. Furthermore, during these practices coaches can expand the offensive skillset for a defensive type of player by creating targeted structured drills that require them to attack the defense.
• For future success, teams need to update their tactics and players’ decision-making processes to fully capitalize on the increasingly diversified roles on the court. For example, targeted shooting drills could be designed by using visual signs (e.g., BlazePod lights) that correspond to different dribbling and shooting combinations. In this way, players can prepare for different tactical situations that require different technical solutions.

## 6 Conclusion

It has increasingly been recognized that the traditional five positions and their definitions may no longer be accurate for the current trends in basketball. This is also reflected in our dataset, where nine positions were provided by the coaching staff through the Federation's official platform instead of the well-known five.

We assumed that there won't be big differences between the traditional positions and the three created statuses (Hungarian-educated, Hungarian U23 youth and import) in terms of offensive efficiency indicators. Our results pointed out that during the position analysis there was a difference between the inside and outside positions through the scoring indicators, in preparation and organizing the offense through the indices that involves assists, turnovers. We also noted important differences within the statuses where the main statement is that the import players had the best results, followed by the Hungarian-educated players and the lowest values belong to the Hungarian U23 players. The import players are the main scorers while the other two statuses are involved in the preparation.

As we collected the data, we bumped into difficulties since the database had player duplications. We solved this difficulty by combining the player's statistics. In addition, we suggest expanding the measures indices so we could get a more complex picture about the players' playing style, true value and performance on the court, a more precise describing of their real positions.

Finally, we should mention that in the current research, we didn't examine the tactical decisions and playbooks of either the coaches or the players.

Future research could extend this work by examining defensive efficiency and tactical decision-making as well as different play-types, which may provide a more comprehensive understanding of how positions and statuses shape overall team performance.

## Data Availability

The raw data supporting the conclusions of this article will be made available by the authors, without undue reservation.
